# Pharmacists’ perceptions of the new pharmaceutical vaccination service in Romania: a comprehensive first two-years evaluation

**DOI:** 10.3389/fphar.2024.1476504

**Published:** 2025-01-07

**Authors:** Corneliu-Florin Buicu, Mihaela-Simona Naidin, Marius Calin Chereches, Marina-Daniela Dimulescu, Adina Turcu-Stiolica

**Affiliations:** ^1^ Department of Public Health, Faculty of Medicine, University of Medicine, Pharmacy, Science, and Technology “George Emil Palade”, Targu Mures, Romania; ^2^ Department of Pharmaceutical Management and Marketing, Faculty of Pharmacy, University of Medicine and Pharmacy of Craiova, Craiova, Romania; ^3^ Drug Industry and Pharmaceutical Management Department, Faculty of Pharmacy, University of Medicine, Pharmacy, Science, and Technology “George Emil Palade”, Targu Mures, Romania; ^4^ Doctoral School, University of Medicine and Pharmacy of Craiova, Craiova, Romania

**Keywords:** vaccination service, community pharmacy, flu vaccination, service authorization, chain pharmacy, independent pharmacy

## Abstract

**Introduction:**

Pharmacy-based vaccination services are now available in 56 countries, including Romania, that started administering the flu-vaccines in the community pharmacies from 2022. Assessing how pharmacists managed this new pharmaceutical service in Romania is the subject of this study.

**Methods:**

A cross-sectional study was conducted among all the pharmacies from Romania that were authorized to provide this service (442 pharmacies, from which 53 were in rural areas). An online survey was created using Google Forms and included 28 items, with 24 closed-ended questions and 4 open-ended questions. The questionnaire covered six sections: General information, Patient perspective, Authorization and training within the pharmacy, Administration of flu vaccine services, Pharmacy logistics, and Staff satisfaction. Descriptive statistics and chi-squared tests were applied.

**Results:**

In total, 180 pharmacists participated (response rate was 41%), and the respondents were the pharmacists who administered flu vaccines in these pharmacies. Among the respondents, 92.8% were from urban community pharmacies, and most of them were from Bucharest (26.1%) and East Romania. 88% of respondents considered that this new service will significantly impact the future increase in vaccine coverage rates in Romania. Regarding patients’ perception of this pharmaceutical service (provided by pharmacists), the vaccination service was evaluated very positively by 63% patients and positively by 18% patients, with statistically different perceptions between the types of the pharmacies (*p* < 0.01). A very positive vaccination evaluation was observed more often among national chain pharmacies (73.1%) rather than among local chain pharmacies (35.9%) or independent pharmacies (36.4%). Regarding logistical barriers, 39% of pharmacies reported no issues with vaccine supply. Moreover, 97% of pharmacies had adequate protective materials to safely administer vaccines. In terms of overall satisfaction, 23% of pharmacists reported being very satisfied, while 39% indicated they were satisfied with the new pharmaceutical service they were providing. The majority (82%) felt that their salaries should be increased related to the vaccination service. Additionally, there is a need for improvements in the pharmacy schedule and the advance scheduling of vaccinations.

**Conclusion:**

This study was developed to assist future health policies through expansion of advanced pharmaceutical services, and adding other vaccines to community pharmacy portfolios.

## 1 Introduction

Worldwide, community pharmacists’ responsibilities have grown to include the authority to administer vaccinations (Le et al., 2022). Pharmacy-based vaccination services are now available in 56 countries worldwide and territories, up from 34 countries in a previous survey from 2020 (International Pharmaceutical Federation, 2024). Before the COVID-19 pandemic, pharmacists were already permitted to give certain vaccines to specific groups, such as the elderly and those at higher risk (Visacri et al., 2021). Additionally, there were differences between countries regarding whether these vaccines could be given with or without a prescription (Duch et al., 2021). Research (Poudel et al., 2019) indicates that pharmacists acting as vaccinators, vaccine advocates, educators, and managing vaccine supplies enhance vaccination coverage and increase the number of people getting vaccinated. Pharmacists are among the most accessible healthcare professionals in the United States (Bach and Goad, 2015). In many other countries, especially developing ones, they are often the first point of contact for individuals lacking access to a primary healthcare provider (Sakeena et al., 2018). Consequently, pharmacists now have greater opportunities to enhance immunization rates and advance public health (Steeb and Ramaswamy, 2019). In Romania, confidence in community pharmacists has been demonstrated to be high, while patient satisfaction with community pharmacy services remains at a moderate level (Manoliu-Hamwi et al., 2024).

Service evaluation studies show high levels of patient satisfaction, highlighting the importance of maintaining pharmacy vaccination services. However, there is a disparity in pharmacists’ willingness to offer these services. In this context, willingness is defined as the proportion of pharmacists willing to provide pharmacy vaccination services. In countries like the United States and Canada, where pharmacy vaccination services are well-established, willingness ranges from 52% to 98% (Zhao et al., 2021). Other studies (Turcu-Stiolica et al., 2021; Balkhi et al., 2018) showed that in countries where pharmacy vaccination services are either new or under consideration, willingness ranges from 45% to 83%. Barriers affecting pharmacists’ willingness to offer pharmacy vaccination services include poorly defined regulations, lack of training, high workload, and inadequate reimbursement. For example, many vaccines provided through pharmacy vaccination services are not reimbursable or covered by health insurance (Alsabbagh et al., 2018). Additionally, inadequate staffing leading to high workloads and limited availability of training or certification programs further hinder pharmacists’ participation in pharmacy vaccination services (Tadele et al., 2023).

In 2021, the Romanian Ministry of Health has defined for the first time eleven new advanced pharmaceutical services that will be provided by the pharmacists in the authorized pharmacies from Romania (New Pharmaceutical Services, 2021). According to this legislation, the pharmacists who wish to provide this pharmaceutical service must obtain a certificate that is valid for 3 years. This certificate is obtained after completing a training course organized by an accredited higher education institution with a human medical-pharmaceutical profile, in collaboration with the Romanian College of Pharmacists, and focused on the specific theme of the respective pharmaceutical service. The course is designed to provide the necessary competencies for delivering the respective pharmaceutical service and concludes with the issuance of a certificate that attests to the appropriate training in this regard. Some of the eleven pharmaceutical services are: SFA01 (The polypharmacy evaluation and monitoring service), SFA02 (The chronic patient management service—for hypertensive, diabetic, pulmonary conditions, and dyslipidemia), SFA03 (The monitoring service for patients undergoing treatment with oral antineoplastic agents), SFA04 (The monitoring service for patients undergoing treatment with oral anticoagulants), SFA05 (The specific monitoring service for patients with chronic obstructive pulmonary diseases). The flu-vaccination service, according to SFA10 service, was the first pharmaceutical service introduced in practice by the Order of Romanian Ministry of Health no. 3262/19.10.2022, which approved the guidelines for providing this service in the community pharmacies from Romania. A very important step in development of this pharmaceutical service was reached through the article no. 153 from HG 839/18.11.2023 which approved reimbursed flu-vaccines with different reimbursement percentages (50%, for adults aged between 45 and 65 without chronic diseases, and 100%, for the other individuals: children between 6 months and 19 years old, pregnant women, people over 65 years of age, specialized medical and healthcare personnel and auxiliary personnel, persons aged more than 19 and less than 65 years with one chronic disease as, for example, chronic cardiovascular disease, chronic respiratory disease, chronic kidney or liver disease, etc.).

Influenza vaccination campaigns are typically seasonal, aligning with the periods of highest risk for influenza outbreaks. In most regions, including Romania, flu vaccination efforts concentrate in the fourth quarter (Q4) of the year, from October to December, just before the peak flu season in winter. This period is critical for maximizing immunity within the population. Additionally, vaccination may continue into the first quarter (Q1) of the following year, particularly for late adopters, vulnerable populations, or those who missed earlier opportunities. These periods are central to planning and evaluating the implementation of vaccination services in community pharmacies.

This current research aims to explore pharmacists’ practices and perceptions regarding vaccination SFA10 service management, focusing on pharmacy authorization, completing the training courses, perceptions of pharmacists and patients on this new pharmaceutical service.

## 2 Materials and methods

### 2.1 Study design

A cross-sectional, online, self-administered, anonymous and confidential survey, was designed via online Google Forms for the pharmacists working in the community pharmacies authorized to perform anti-flu vaccination after legal approval of this service, October 2022. To gather responses, we invited all pharmacies authorized to administer influenza vaccines to participate in our questionnaire. This was accomplished through outreach to national and regional pharmacy chains, independent pharmacies, and the Chamber of Pharmacists. The invitation was disseminated widely to ensure inclusivity across all eligible pharmacies. The questionnaire was distributed online, with participation on a voluntary basis. To enhance the response rate, multiple reminders were sent throughout the data collection period. This strategy ensured that the sample represented a diverse range of perspectives from pharmacists actively engaged in delivering the new vaccination service. The survey was disseminated among the 21 counties from Romania, and Bucharest capital. There were 442 pharmacies in Romania that were allowed to administer anti-flu vaccines at the start of our investigation, 20 February 2024. Of these, 239 (54%) are associated with national pharmacy chains (pharmacy networks that operate across entire country with extensive geographical coverage), 147 (33%) with regional chains or groups of pharmacies (smaller pharmacy networks that operate within a specific region or locality, serving a limited geographical area), and 56 (13%) as standalone independent pharmacies (independently owned and operated pharmacies, not part of any larger chain). At the beginning of the study, 53 (12%) of the 442 pharmacies authorized to administer influenza vaccines were located in rural areas. To gather as many responses as possible, emails were sent to pharmacy associations, national or regional chain management, pharmacist groups, the Chamber of Pharmacists, and other relevant pharmacy associations. It's important to note that the questionnaires did not collect any personal information, thus the identity of the respondents remains anonymous.

The current study presents the results of the questions about perceptions of pharmacists about the new vaccination service. The study adhered to the CHERRIES (Checklist for Reporting Results of Internet E-Surveys) guidelines to ensure methodological rigor and transparency in designing and reporting the online surveys (Supplementary Table 1). The questionnaire was developed using information gathered from anti-flu vaccination literature available in pharmacies. The questionnaire was designed by a team of experts, including pharmacists with practical experience in vaccination services (M.-S.N. and M.C.C.) and researchers with expertise in survey design (A.T.-S.). The goal of the survey was to involve as many community pharmacies as possible with vaccination service authorization in the identification of the current practices and future needs. It consisted of 28 items, with 24 closed-ended questions (these provided structured response options for quantitative analysis) and 4 open-ended questions (these allowed participants to share additional perspectives and provide qualitative insights). The questionnaire covered six sections (Supplementary Table 2):

• General information (capturing demographic and operational data of participating pharmacies),
• Patient perspective (understanding pharmacists’ perceptions of how patients evaluated the vaccination services),
• Authorization and training within the pharmacy (assessing the authorization process and training needs for pharmacy personnel),
• Administration of flu vaccine services (evaluating the practical aspects of vaccine administration, including workflow and protocols),
• Pharmacy logistics (examining resources, infrastructure, and logistical challenges associated with providing vaccination services), and
• Staff satisfaction (exploring the attitudes and satisfaction of pharmacy staff regarding their involvement in vaccination services).

Prior to distribution, a brief pilot study was conducted to ensure that the questions were well-constructed and easily understood.

The protocol was approved by the Ethics Committee of the University of Medicine and Pharmacy of Craiova (no. 70/29.01.2024) according to the requirements of the Helsinki Declaration. All the participants provided electronic informed consent responding to the first question of the survey. The study was performed between 20 February 2024 and 25 March 2024.

### 2.2 Statistical analysis

Statistical analysis of the data from the survey consisted mainly of a descriptive analysis (i.e., frequencies and percentages). Descriptive statistics were generated as a total and by type of community pharmacy: national chain pharmacy, local chain pharmacy and independent pharmacy. Differences between the three types of community pharmacies were analyzed with Chi squared test. We applied Benjamini–Hochberg correction to adjust the significance threshold (α_adjusted_), mitigating the risk of Type I and Type II errors, using the p.adjust () function in R with the method set to “BH.” The open-ended responses were analyzed using thematic analysis, following Braun and Clarke’s framework (Braun and Clarke, 2006), to identify, organize, and interpret key themes. The analysis was conducted systematically by two independent researchers (A.T.-S. and M.C.C.) who reviewed and coded the data, resolving discrepancies through discussion and consensus. All analysis will be performed using R packages (R Core Team, 2022). Statistical significance was established at *p* < 0.05.

## 3 Results

### 3.1 Sample characteristics of the pharmacies

A total of 180 pharmacies participated in the study, with demographic information provided in Table 1. Among the respondents, 92.8% were from urban community pharmacies, and most of them were from Bucharest (26.1%). As at the time of the survey distribution there were 442 authorized community pharmacies to administer flu-vaccines, this leads to a study response rate of 40.7% (from which 54.4% form national chain pharmacies, 26.5% from local chain pharmacies, and 19.6% from independent pharmacies). Most pharmacies were from Bucharest (26.1%) and East Romania: Galati (12.8%), Iasi (12.2%), Constanta (7.8%).

**TABLE 1:** Demographics of survey participants (community pharmacies).

	Pharmacies (n = 180)
Type	
National chain pharmacy	130 (72.2%)
Local (regional chain pharmacy)	39 (21.7%)
Independent pharmacy	11 (6.1%)
Area of activity	
Urban	167 (92.8%)
Rural	13 (7.2%)
County	
Bucharest	47 (26.1%)
Galati	23 (12.8%)
Iasi	22 (12.2%)
Constanta	14 (7.8%)
Brasov	9 (5%)
Other	10 (5.6%)

Across the three flu vaccination periods (Q4 2022, Q1 2023, and Q4 2023), the majority of vaccinations were anticipated to occur in Q4 2023 (82%), compared to significantly lower proportions in Q4 2022 (4%) and Q1 2023 (14%).

**FIGURE 1:**
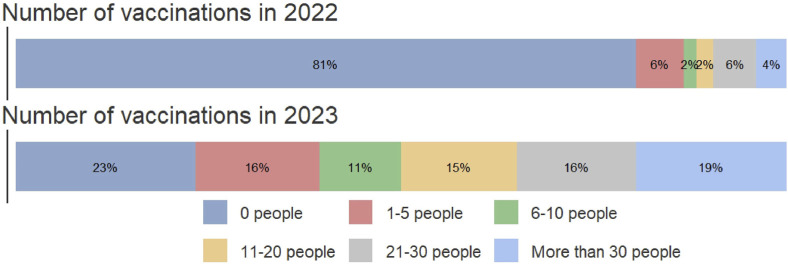
Number of flu-vaccinations in 2022 and 2023 in the community pharmacies from Romania.

A higher number of vaccinations were administered in 2023 compared to 2022. Specifically, more than 30 people were vaccinated in 19% of pharmacies in 2023, compared to only 4% in 2022. Similarly, 21–30 individuals were vaccinated in 16% of pharmacies in 2023, compared to 6% in 2022, and 11–20 people were vaccinated in 15% of pharmacies in 2023, compared to 2% in 2022, as shown in Figure 1. Additionally, a greater proportion of pharmacies administered higher numbers of vaccinations in 2023 (*p* < 0.01). Additionally, 88% of pharmacists believed that offering flu vaccinations in community pharmacies will lead to an increase in the number of people getting vaccinated in the future.

### 3.2 Patients’ perceptions regarding the pharmaceutical service of vaccination

Regarding how pharmacists perceived patients’ evaluations of the vaccination service, patients rated the service very positively in 113 cases (63%) and positively in 33 cases (18%). The primary reasons patients chose to vaccinate in the pharmacy included the quick service (62%) and trust in the pharmacist (57%). Additionally, convenience was a notable factor, cited by 67 respondents (37%).

Patients considered that “pharmacists have more time to discuss the importance of vaccination compared to medical offices, which are extremely busy during the vaccination period. As a result, patients leave the pharmacy with a wealth of new information.” They also found it advantageous that the entire family could be vaccinated at once. Additionally, the extended hours of the pharmacy, its high accessibility, and its proximity to their homes were significant benefits.

We found that urban/rural environments affected patient satisfaction (χ2 = 16, *p* = 0.001) with patients vaccinated in the urban pharmacies being more satisfied than patients vaccinated in the rural pharmacies (very positive and positive, 82.6% vs. 72.7%).

**FIGURE 2:**
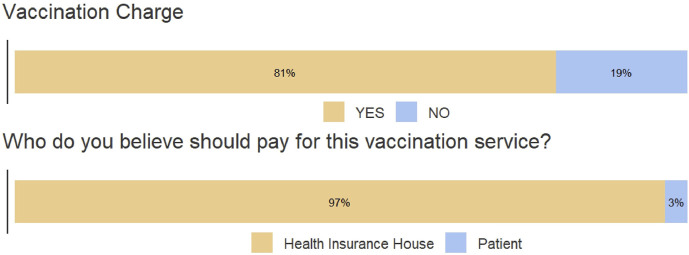
Respondents’ perceptions on the vaccination charge.

The vaccination service provided in community pharmacies can be free of charge or for a fee, according to the law (New Pharmaceutical Services, 2021). The manager of the pharmacy determines whether vaccination services are offered free of charge or for a fee. In the latter case, the vaccination service fee cannot exceed the rate approved by the Order of the Minister of Health No. 964/2022 regarding the approval of the Technical Norms for the implementation of national public health programs, with subsequent amendments and additions (Technical Norms National Public Health Programs, 2022). Among the pharmacies included in the study, 154 (86%) administered vaccines free of charge, while the rest charged between 20 RON (4 EUR) and 54 RON (11 EUR). The respondents believed that the vaccination service should be paid (81%), and most of them (97%) stated that the Health Insurance House should cover the cost, as shown in Figure 2.

### 3.3 Authorization and training of pharmacists for providing the vaccination service

The pharmacies obtained the authorization to conduct seasonal flu vaccination activities from the Romanian Ministry of Health. The process was considered rather bureaucratic by 59% and simple by 22%, with most of the cases (51%) taking less than 1 month to obtain the authorization. Regarding the mandatory course on vaccination for pharmacists who agree to administer flu-vaccines, most of them considered it very efficient (52%) and efficient (46%).

### 3.4 Organization of the vaccination service in the community pharmacy

**FIGURE 3:**
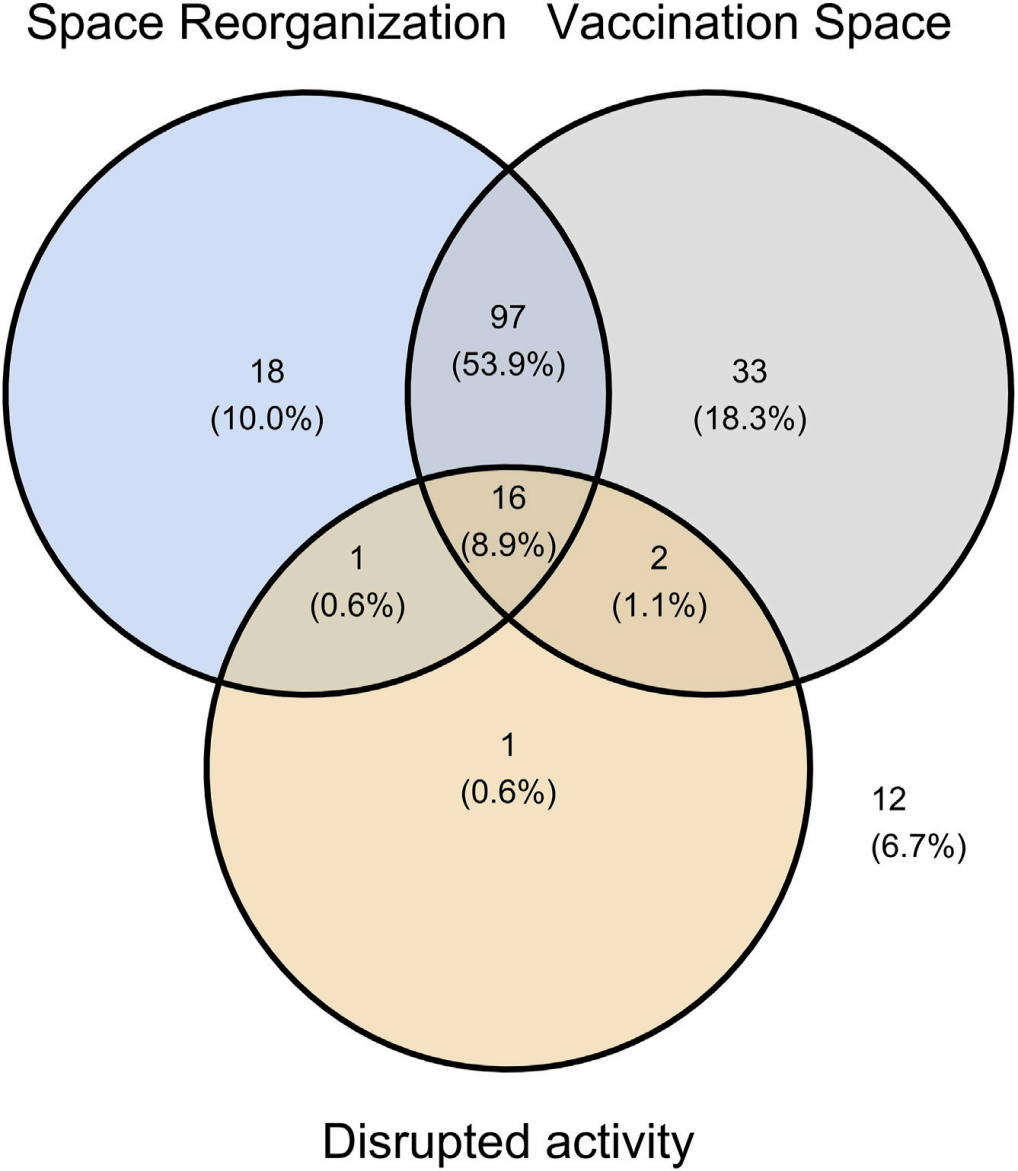
Venn diagram illustrating the logical relationship between space reorganization of the community pharmacy, vaccination space and disrupted activity by the new vaccination service. Space reorganization (yes/no), Vaccination space (Vaccination room/Pharmacist chief room), Disrupted activity (Not significant/Significant).

Pharmacies must organize a designated space for vaccination and post-vaccination monitoring. The vaccination space could be organized either in the pharmacist’s chief room or in a newly designated vaccination room. Most pharmacies (73%) reorganized their space, and the vaccination service is conducted in a specially designed room equipped for vaccination in 82% of the pharmacies (named “vaccination room”). It was found that normal activity was not disrupted at all in 47% of the pharmacies and was moderately disrupted in 42% of the pharmacies. After analyzing the Venn Diagram (with “vaccination space” selected as “Vaccination room,” “Space reorganization” as “Yes,” and “Disrupted activity” as “Significant”), we observed that pharmacy activity was considered significantly disrupted in most cases when the pharmacy was reorganized to include a vaccination room (Figure 3).

In 88% of the pharmacies, it was not considered necessary to have a different vaccination schedule, and patients had the opportunity to make an appointment for vaccination in 88% of the pharmacies.

### 3.5 Logistical barriers

Regarding logistical barriers, 39% of pharmacies reported no issues with vaccine supply, while 41% experienced occasional vaccine supply problems. Additionally, 97% of pharmacies had sufficient protective materials to safely administer the vaccines.

Overall, 23% of the pharmacists were very satisfied and 39% were satisfied with the new pharmaceutical service. Most of them (82%) believed their salary should be increased. Additionally, the pharmacy schedule and the advance scheduling of vaccinations need to be improved.

### 3.6 Results of comparisons among the groups of pharmacies

Upon monitoring the differences among the three types of community pharmacies, we observed the chain pharmacies (national or regional) were more likely to introduce this new pharmaceutical service in urban environments compared to independent pharmacies (but not statistically significant after the Benjamini–Hochberg correction), as shown in Table 2. Most of the respondents were from the national chain pharmacies from Bucharest, and from East Romania regarding the regional chain and independent pharmacies (p < α_adjusted_). All types of the pharmacies started to vaccinate more in Q4 2023 (*p* = 0.43), but most of the vaccinations were provided by the national chain pharmacies (p < α_adjusted_).

**TABLE 2:** Comparison among the three types of pharmacies: national pharmacy chain, regional pharmacy chain and independent pharmacy.

	National chain pharmacy (N = 130)	Regional chain pharmacy (N = 39)	Independent pharmacy (N = 11)	*p*-value
Environment				0.03
Rural	7 (5.4%)	3 (7.7%)	3 (27.3%)	
Urban	123 (94.6%)	36 (92.3%)	8 (72.7%)	
County				<0.01*
Bucharest	44 (33.8%)	3 (7.7%)	0	
Galati	11 (8.5%)	8 (20.5%)	3 (27.3%)	
Iasi	11 (8.5%)	6 (15.4%)	0	
Constanta	8 (6.2%)	0	1 (9.1%)	
Brasov	7 (5.4%)	15 (38.5%)	2 (18.2%)	
Other	49 (37.7%)	7 (18%)	5 (45.5%)	
Vaccination period				0.42
Q4 2022	4 (3.1%)	3 (7.7%)	1 (9.1%)	
Q1 2023	19 (14.6%)	6 (15.4%)	0	
Q4 2023	107 (82.3%)	30 (76.9%	10 (90.9%)	
Number of vaccinations 2022				0.93
0 people	102 (78.5%)	34 (87.2%)	9 (81.8%)	
1–5 people	9 (6.9%)	1 (2.6%)	1 (9.1%)	
6–10 people	2 (1.5%)	1 (2.6%)	0	
11–20 people	4 (3.1%)	0	0	
21–30 people	7 (5.4%)	2 (5.1%)	1 (9.1%)	
More than 30 people	6 (4.6%)	1 (2.6%)	0	
Number of vaccinations 2023				<0.01*
0 people	19 (14.6%)	20 (51.3%)	3 (27.3%)	
1–5 people	18 (13.8%)	8 (20.5%)	3 (27.3%)	
6–10 people	14 (10.8%)	4 (10.3%)	1 (9.1%)	
11–20 people	22 (16.9%)	3 (7.7%)	2 (18.2%)	
21–30 people	23 (17.7%)	3 (7.7%)	2 (18.2%)	
More than 30 people	34 (26.2%)	1 (2.6%)	0	
Vaccination evaluation by patients				<0.01*
Negative	0	1 (2.6%)	0	
Neutral	14 (10.8%)	16 (41.0%)	3 (27.3%)	
Positive	21 (16.2%)	8 (20.5%)	4 (36.4%)	
Very positive	95 (73.1%)	14 (35.9%)	4 (36.4%)	
Vaccination charge, yes	28 (21.5%)	5 (12.8%)	2 (18.2%)	0.48
Who do you believe should pay for this vaccination service?				0.67
Patient	4 (3.1%)	2 (5.1%)	0	
National Health Insurance House	126 (96.9%)	37 (94.9%)	11 (100%)	
How long did the authorization process take?				<0.01^†,^*
Less than 1 month	76 (58.5%)	10 (25.6%)	6 (54.6%)	
1–2 months	43 (33.1%)	18 (46.2%)	4 (36.4%)	
More than 2 months	11 (8.5%)	11 (28.2%)	1 (9.1%)	
The complexity of the authorization process				0.28
Rather bureaucratic	80 (61.5%)	21 (53.8%)	6 (54.6%)	
Simple and easy	30 (23.1%)	6 (15.4%)	3 (27.3%)	
Very complex	20 (15.4%)	12 (30.8%)	2 (18.2%)	
Postgraduate training course				0.75
Very efficient	68 (52.3%)	22 (56.4%)	4 (36.4%)	
Efficient	59 (45.4%)	16 (41.0%)	7 (63.6%)	
Inefficient	3 (2.3%)	1 (2.6%)	0	
Space reorganization, yes	98 (75.4%)	29 (74.4%)	5 (45.5%)	0.10
Vaccination space				0.01*
Vaccination room	114 (87.7%)	27 (69.2%)	7 (63.6%)	
Pharmacist chief room	16 (12.3%)	12 (30.8%)	4 (36.4%)	
Disrupted activity				0.59
Not at all	56 (43.1%)	21 (53.8%)	7 (63.6%)	
Moderate	59 (45.4%)	14 (35.9%)	3 (27.3%)	
Significant	15 (11.5%)	4 (10.3%)	1 (9.1%)	
Different vaccination schedule, Yes	14 (10.8%)	5 (12.8%)	3 (27.3%)	0.27
Patient’s opportunity to make an appointment for the vaccination, Yes	119 (91.5%)	29 (74.4%)	10 (90.9%)	0.02*
Do you think this pharmaceutical service will increase the number of people getting vaccinated in the future? Yes	115 (88.5%)	33 (84.6%)	10 (90.9%)	0.77
Did you have vaccine supply problems?				0.15
No	51 (39.3%)	12 (30.8%)	8 (72.7%)	
Sometimes	55 (42.3%)	17 (43.6%)	2 (18.2%)	
Yes	24 (18.5%)	10 (25.6%)	1 (9.1%)	
Did you have enough protective materials to administer the vaccines safety? Yes	126 (96.9%)	37 (94.9%)	11 (100%)	0.67

*statistically significant after applying the Benjamini–Hochberg correction. †statistically significant at 0.05.

National chain pharmacies have a substantial market presence in Bucharest, representing 34% of the authorized pharmacies providing vaccines that responded to our survey. Additionally, certain counties are dominated by regional chain pharmacies, such as in Galați (15%) and Constanța (39%), or by independent pharmacies, such as in Iași (27%).

Regarding how pharmacists perceived patients’ evaluations of the vaccination service, the vaccination service was evaluated very positively by 113 patients (63%) and positively by 33 patients (18%), with statistically significant differences in perceptions between types of pharmacies (p < α_adjusted_). A very positive evaluation of the vaccination service was more common among patients of national chain pharmacies (73.1%) compared to those of local chain pharmacies (35.9%) or independent pharmacies (36.4%).

Regarding the authorization duration, it seems that the process varies depending on the type of the pharmacy (*p* < 0.01). Our survey found that 58% of pharmacists in national chain pharmacies reported an authorization process of less than 1 month, comparable to the responses from independent pharmacies. In contrast, regional pharmacies experienced longer wait times, with only 26% completing the process in under 1 month. A percent of 73% of independent pharmacies did not mention supply issues, unlike national or regional chains (39% or 31%, respectively).

Table 2 summarizes the comparison among the three types of pharmacies: national pharmacy chain, regional pharmacy chain and independent pharmacy.

## 4 Discussion

### 4.1 Role of pharmacies in expanding healthcare services

As pharmaceutical services expand in the world, the frequency of vaccinations administered by specialized pharmacists in community pharmacies also increases in Romania. This study explored the perceptions of pharmacists regarding the implementation of this service in community pharmacies in Romania. We identified the limitations that pharmacists believe need to be addressed to enhance this pharmaceutical service.

### 4.2 Pharmacists’ perspectives on the vaccination service

We evaluated responses of 54% national chains pharmacies, 33% regional pharmacies, and 13% independent pharmacies. The percentages have relevance because national chains typically have the resources and infrastructure to implement new services like vaccinations quickly and consistently, whereas regional chains and independent pharmacies may face unique challenges or opportunities in adopting vaccination services. Regarding the volume of vaccinations in 2023, 61% of pharmacies belonging to national chains have vaccinated more than 10 individuals. This could be attributed to their ability to attract more customers and perhaps more aggressive marketing tactics associated with national chains. It’s worth noting that a higher percentage of independent pharmacies (36%) outperformed regional chains (19%) in terms of vaccinating more than 10 individuals. This could be due to the direct involvement of the independent pharmacy owners. This indicates that independent pharmacies are motivated to offer vaccination services as it can lead to increased business. A meta-analysis conducted in 2023 on pharmacy-based immunization concluded that most studies focused on pharmacy chains rather than independent pharmacies. Additionally, several pharmacy-based immunization strategies have emerged, aiming to leverage the potential of pharmacies as immunization centers (Romero-Mancilla et al., 2023).

### 4.3 Patient perceptions and benefits of vaccination in pharmacy

In our study, the flu vaccination service was evaluated very positively (63%) and positively (18%) by the patients, only 1% have negative perceptions and 18% neutral perceptions (pharmacists perceived patients’ evaluations of the vaccination service). We observed that 73% of national chain pharmacies indicated that patients responded “very positively” to the flu vaccination. The other two categories had a more balanced evaluation of patient responses. It seems that the staff at national chain pharmacies may have a subjective view due to being appraised on similar metrics and thus being predisposed to self-evaluate in this manner. Manoliu-Hamwi et al. (2024) measured patient satisfaction with the quality of community services in Romania, but without being specifically on vaccine services, even if they applied a questionnaire to measure the patient satisfaction between January and June 2023. Overall patient satisfaction with community services was moderate; however, confidence in these services was rated highly (Manoliu-Hamwi et al., 2024). The level of satisfaction was influenced by the urban/rural environment, the same we found in our study. We found that urban/rural environments affected patient satisfaction with patients vaccinated in the urban pharmacies being more satisfied than patients vaccinated in the rural pharmacies.

The study we conducted provided information on the patients coming to the community pharmacies to be vaccinated on the recommendations of the family doctor, among which we identified the advantages of vaccinating the entire family at once. We also identified the benefits of the pharmacy’s extended hours, high accessibility, and proximity to home, which lead to better pharmacist-patient communication and increased patient trust in the pharmacist. Patients’ perception of vaccination in the pharmacy was reported as a great benefit due to the time saved compared to the same services offered in an outpatient clinic or in a doctor’s office (Kowalczuk et al., 2022). Another team of researchers has also demonstrated the time saved by patients and their preference to be vaccinated in the community pharmacy, even though this would sometimes entail additional costs compared to scheduling vaccinations at the hospital (Anderson and Thornley, 2014). As in previous studies made in England and Poland (Kowalczuk et al., 2022; Anderson and Thornley, 2014), the main reasons why patients choose to get vaccinated in community pharmacies in Romania were convenience, quickness and time saving. In addition, a very high percentage also reported their confidence in the pharmacist.

There were recorded different patient perceptions of vaccination in community pharmacies in the literature, mostly positive. One of the patients involved in the study mentioned that the patients need this vaccination service because it is much easier for them to reach community pharmacies which are numerous in a city and close to them, plus they do not have to make an appointment, take a day off work, wait in crowded spaces or face situations where the hospital is out of stock and they have to reschedule their appointments (Al Aloola et al., 2020; Alnahar et al., 2022). Another patient expressed his opinion about the ability of pharmacists to educate the population about immunization and its importance. Another participant asserted that when a healthcare professional, a nurse, for example, administers the vaccine, it should be supervised by a pharmacist, because it is the pharmacist who decides what type of vaccine should be given to the patient (Al Aloola et al., 2020).

Results of our study showed 88% of pharmacists believed that offering flu vaccinations in community pharmacies will lead to an increase in the number of people getting vaccinated in the future. Pharmacists play an increasingly significant role in public health initiatives, particularly in the realm of vaccination programs. As highlighted in recent literature, community pharmacists have emerged as key providers of vaccine information and administration, especially for influenza immunization (Lim et al., 2023). This positioning aligns with the broader recognition of pharmacy’s potential to enhance primary care services (Kirkdale et al., 2017a). Indeed, there is growing evidence to suggest that expanding pharmacists’ involvement in antimicrobial stewardship and vaccination services could yield substantial benefits. Such expansion not only supports the optimal use of antimicrobials but also presents an opportunity to improve vaccination coverage across populations (Lim et al., 2023; Parajuli et al., 2022). By leveraging their accessibility and expertise, pharmacists are well-positioned to address barriers to vaccination and contribute meaningfully to public health strategies aimed at increasing immunization rates.

The majority of patients (88%) interviewed in Houle’s study agreed that vaccination should be cost-effective (Houle et al., 2019), and similar data were obtained in the study we conducted. Among the subjects, 80.6% recognized the need to implement vaccination services costs in Romania. Also, some patients claim that vaccinating in a pharmacy versus a hospital is cost-saving. One of the subjects stated that in the pharmacy they only pay for the vaccine, without any additional fees, which is a benefit compared to vaccination in a hospital clinic (Nusair et al., 2020).

### 4.4 Challenges and barriers to implementation

It seems that the authorization process for Romanian pharmacies to be allowed to administer the vaccines varies depending on the type of pharmacy. According to our survey, 58% of pharmacists in national chain pharmacies reported that the process took less than 1 month, which is similar to the responses from independent pharmacies. However, regional pharmacies experienced longer wait times for authorization (26% for less than 1 month). National chains seemed to have a designated regulatory department responsible for preparing the necessary documentation and procedures, which likely expedited the process. Independent pharmacies also showed a strong willingness to allocate time and resources to expedite the authorization process. In contrast, regional chains may have faced delays due to limited resources to handle multiple authorizations simultaneously.

In our study we identified the need for a dedicated space for vaccination. Specifically, 73.3% of the pharmacists included in the study reported reorganizing the space to facilitate the service, and 82.2% indicated that the vaccination area was located in a separate room from the head pharmacist’s office. Similarly, participants in other studies we analyzed also emphasized the importance of a dedicated vaccination area in community pharmacies to ensure confidentiality and patient safety. Such a space should include a sterile area and a refrigerator for storing vaccines (Al Aloola et al., 2020).

Studies and practice have shown that pharmacists are trusted healthcare professionals (Tareq et al., 2021) and some participants stated that the implementation of vaccination in community pharmacies and increased patient trust in pharmacists would also improve the counseling and pharmacist-patient relationship (Nusair et al., 2020).

Interestingly, 73% of independent pharmacies reported no supply issues, in contrast to national or regional chains, which reported such issues at rates of 39%, and 31%, respectively. Given the specific structure of the Romanian market, which is dominated by vertically integrated chains, we initially expected the opposite. We hypothesize that independent pharmacies obtaining authorization had carefully assessed vaccine availability well in advance and were fully prepared for this business endeavor. In contrast, pharmacies within national or regional chains likely entered the program following general management decisions, with supply chain procedures not yet fully adapted for the first flu vaccination season.

We observed more than half of the pharmacists were very satisfied (23%) and satisfied (39%) with the new vaccination service in our study. The reported satisfaction levels should be interpreted in the context of implementing a novel pharmaceutical service that necessitates significant professional and logistical adaptation. A notable finding of this study is that 82% of pharmacists that provide vaccination services expressed the view that their salaries should be increased, which likely reflects their perception of the additional workload and responsibilities associated with vaccination services, relative to their current remuneration. Several factors appear to underlie this sentiment. Pharmacists often perceive their salaries as insufficient relative to their qualifications and workload. The introduction of vaccination services, which generates a new revenue stream for pharmacies, is viewed by many as an opportunity to align their compensation with the increased demands of their role. Furthermore, the process of obtaining authorization for vaccination services and reorganizing pharmacy spaces to meet operational and regulatory requirements imposes considerable demands in terms of time, effort, and financial resources. These challenges contribute to the perception that enhanced compensation is warranted to account for these additional commitments. Additionally, the delivery of vaccination services requires a greater degree of patient engagement, particularly in educating individuals about the benefits and importance of immunization. This expanded role demands significant time and the application of specialized expertise, further intensifying pharmacists’ workload and professional responsibilities.

These findings highlight the importance of addressing pharmacists’ perspectives on compensation and workload to ensure their continued engagement and the sustainability of vaccination programs within community pharmacy settings. Additionally, improvements are needed in the pharmacy schedule and the advance scheduling of vaccinations. The level of pharmacist satisfaction with flu vaccination services is associated with various factors such as payment, workplace stress, and level of experience, according to recent studies conducted in Australia (Shahin et al., 2024).

### 4.5 Future developments and policy recommendation

Another aspect we concluded was that the pharmacists considered that the promotion of the influenza vaccination service in the pharmacy should be carried out through additional communication channels, with the possibility of displaying the locations where vaccinations are provided on the company’s website and in the pharmacy windows. The list of vaccination points should be maintained and updated weekly both on the College’s website and on the Ministry of Health’s website. A well-defined and standardized credentialing process at the national level (not local, with varying rules from county to county) is essential as well. Pharmacies have become increasingly involved in flu vaccination campaigns, reflecting their expanding role as primary healthcare providers. This can be attributed to the implementation of regulatory frameworks enabling pharmacist-led influenza vaccination, as well as the development of appropriate training programs and operational procedures (Kirkdale et al., 2017b).

This study provides valuable insights into pharmacists’ perceptions of the newly implemented pharmaceutical vaccination service in Romania, highlighting both its strengths and areas for improvement. To ensure the successful development of the service, clear guidelines and policies should be established to ensure fair remuneration for pharmacists providing vaccination services. Adequate compensation would not only reflect the added responsibilities but also motivate pharmacists to actively engage in the service. Regarding the logistical barriers, stakeholders should collaborate to streamline vaccine procurement and distribution processes, ensuring consistent and timely supplies across all types of community pharmacies. This could involve partnerships between government health authorities and industry to mitigate supply chain disruptions.

### 4.6 Limitations of the study

A limitation of our study is regarding the potential for bias in the reporting of patient satisfaction. In our study, the data on patient perceptions of the pharmaceutical vaccination service were indeed reported by pharmacists based on their interactions with patients and feedback received during the service. While this approach provides valuable insights into how pharmacists perceive patient satisfaction, we acknowledge that it may introduce a degree of bias, as the data reflect pharmacists’ interpretations rather than direct patient responses. To address this, we highlight the need for future studies to include direct patient surveys or other objective measures to validate and complement the findings, especially after more vaccinations will be provided in the community pharmacies from Romania.

Another limitation of our study is its cross-sectional survey design, which provides a snapshot of pharmacists’ attitudes and experiences at a specific point in time but does not allow for capturing changes or developments over time. A longitudinal follow-up study would be particularly valuable for examining how pharmacists’ perceptions and experiences evolve with increased exposure and practice in administering vaccines. Future research should prioritize this approach to provide deeper insights into long-term trends, enabling a more comprehensive understanding of the dynamics and impacts of integrating vaccination services into pharmacy practice.

## Data Availability

The raw data supporting the conclusions of this article will be made available by the authors, without undue reservation.
