# Supplementary material for: Pharmacists’ perceptions of the new pharmaceutical vaccination service in Romania: a comprehensive first two-years evaluation
Source: Front Pharmacol. 2025 Jan 7;15:1476504. doi: 10.3389/fphar.2024.1476504 (PMC11747464; doi:10.3389/fphar.2024.1476504)
Supplement: Supplementary file 2 [file Table2.docx]

Supplementary Material

**Supplementary Table S2.** Structure of the questionnaire.

| **Outcome** | **Variable** | **Methods of Analysis** |
| --- | --- | --- |
| **Pharmacy type, location, and county** | What is the type of the pharmacy?   - National Pharmacy chain - Local (regional) pharmacy chain - Independent pharmacy | Percentages and frequencies for categories |
|  | Where is located the pharmacy?   - Urban - Rural |  |
|  | What county is this pharmacy located in? |  |
|  | How many people have been vaccinated in 2022?   - 0 people - 1-5 people - 6-10 people - 11-20 people - 21-30 people - More than 30 people |  |
|  | How many people have been vaccinated in 2023?   - 0 people - 1-5 people - 6-10 people - 11-20 people - 21-30 people - More than 30 people |  |
| **Patients’ perceptions** | How did patients evaluate the vaccination service at your pharmacy?   - Very positive - Positive - Neutral - Negative |  |
|  | What were the main reasons patients choosing to get vaccinated at the pharmacy?   - Collusion - Rapidity - Trust in the pharmacist - Other |  |
|  | Do you believe that vaccination administration should be subject to a fee? (Yes/No) |  |
|  | If you answered "YES" to the previous question, who do you believe should pay for this vaccination service?   - Health Insurance House - Patient |  |
|  | What price did patients pay for the flu vaccination service? |  |
| **Authorization and training of pharmacists for providing the vaccination service.** | How long did the authorization process take to be able to administer vaccines?   - Less than 1 month - 1-2 months - More than 2 months |  |
|  | How do you rate the complexity of the authorization process?   - Rather bureaucratic - Simple and easy - Very complex |  |
|  | After completing the postgraduate training course, you considered staff training to be:   - Very efficient - Efficient - Inefficient |  |
| **Organization of the vaccination service in the pharmacy.** | Was it necessary to reorganize the space to administer the vaccines? (Yes/No) |  |
|  | The vaccination space is:   - Chief pharmacist's office - Vaccination room |  |
|  | How has the normal activity of the pharmacy been disrupted by the vaccination program?   - Significant - Moderate - Not at all |  |
|  | Did you have a different vaccination schedule than the pharmacy's opening hours? (Yes/No) |  |
|  | Did the patients have the opportunity to make an appointment in advance for the vaccination service? (Yes/No) |  |
|  | Do you think that community pharmacy flu vaccination will increase the number of people getting vaccinated in the future? (Yes/No) |  |
| **Logistical barriers** | Have you had problems with the supply of vaccines?   - Yes - Sometimes - No |  |
|  | Did you have enough protective materials to administer the vaccines safely? (Yes/No) |  |
|  | If you answered "No" to the previous question, please specify the problems encountered. |  |
| **Satisfaction of the pharmacists** | How satisfied is the staff with the implementation of this program?   - Very satisfied - Satisfied - Neutral - Dissatisfied - Very dissatisfied |  |
|  | What aspects do you believe could be improved in the vaccination program?   - Salary - Work schedule - Advance scheduling - Other |  |
|  | If you selected "Other" for the previous question, please specify your suggestions. |  |
